# Bone density in transfusion dependent thalassemia patients in Urmia, Iran

**Published:** 2014-04-20

**Authors:** N Valizadeh, F Farrokhi, V Alinejad, SM Said Mardani, N Valizadeh, S Hejazi, M Noroozi

**Affiliations:** 1 Assistant professor of Hematology/Medical Oncology, Urmia University of Medical Sciences, Urmia, Iran; 2 Medical Student, Urmia University of Medical Sciences, Urmia, Iran; 3 MSc. of Biostatistics, Patient Safety Research Center, Urmia University of Medical Sciences, Urmia, Iran; 4 Assistant professor of Rheumatology, Urmia University of Medical Sciences, Urmia, Iran; 5 Assistant professor of Endocrinology and Metabolism, Urmia University of Medical Sciences, Urmia, Iran; 6 Assistant professor of Pediatric Hematology/Medical Oncology, Department of pediatric hematology, Motahari hospital, Urmia university of medical sciences, Urmia , Iran; 7 Assistant professor of Pediatric Hematology/Medical Oncology, Department of pediatric hematology, Motahari hospital, Urmia University of medical sciences, Urmia, Iran.

**Keywords:** Thalassemia, Bone mineral density, Osteoporosis, Bone Loss

## Abstract

**Background:**

Patients with thalassemia major and intermedia are susceptible to osteopenia and osteoporosis. The mechanism of osteoporosis in these patients is multifactorial. Transfusion related iron overload in endocrine organs leads to impaired growth hormone secretion, diabetes mellitus, hypothyroidism, hypoparathyroidism, lack of sex steroids and vitamin D deficiency that contribute to impairment in achieving an adequate bone mass .The aim of this study was assessment of frequency of bone loss in patients with thalassemia major and intermedia in Urmia City of West Azerbaijan, Iran

**Materials and Methods:**

In this cross sectional descriptive study,10 patients (lower than 18 y/o)with transfusion dependent thalassemia attending to Motahari and Emam Khomeini hospitals in Urmia city of Iran were enrolled and scanned for Bone Mineral Density (BMD) starting at around 10 years old.

**Results:**

Tenatients (6 male and 4 female) with transfusion dependent thalassemia (β-thalassemia major and intermedia) aged 13to 17 years in Urmia city of Iran were enrolled. Mean age of patients was 15.1±.37year old. Among them, 8 patients (80%)had low BMD and2 of them (20%) had normal BMD in lumbar spine. Only 30% of patients had low BMD in the neck of femur.

**Conclusion:**

We should perform annual BMD in patients with thalassemia major and intermedia and hemoglobin H disease in age of higher than 8 year old and treat low BMD with administration of bisphosphonate, calcium and vitamin D supplements. Medical consultation with a rheumatologist and /or an endocrinologist should be performed in these patients. Changing lifestyle with mild daily exercise, adequate calcium containing foods, avoiding heavy activities, stop smoking, iron chelation therapy in adequate dosage, early diagnosis and treatment of endocrine insufficiency and regular blood transfusions can help to achieve an optimal bone density in these patients.

## Introduction

Patients with transfusion dependent thalassemia are at risk to osteopenia and osteoporosis.The pathogenesis of osteoporosis in this group is multifactorial and is included environmental, acquired and genetic factors. Transfusion related iron deposition in endocrine glands can cause impaired growth hormone(GH) secretion , lack of sex steroids, diabetes mellitus, hypothyroidism, hypoparathyroidism and vitamin D deficiency and lead to bone loss. Uncontrolled erythropoiesis and progressive marrow expansion are other causes of osteoporosis in thalassemia major and intermedia patients.Osteoporosis is a side effect of iron toxicity on osteoblasts and also Deferoxamine, an iron chelator which is used in these patients. (1-13).

A study established reduced bone formation rate in Thalassemia major patients (14).

The aim of this study was to assess the frequency of bone loss in patients with thalassemia major and intermedia inUrmia city of West Azerbaijan, Iran.

Patients were scanned for bone mineral density (BMD) at anteroposterior lumbar spine (L1-L4) and femoral neck, using dual energy X-ray absorptiometry. The results of a bone density test are presented as a T or a Z score. T-score is comparison of the bone density with what is normally expected in a healthy young with same sex and Z-score is the number of standard deviations above or below what is normally expected for someone with same age, sex, weight, and ethnic origin.

The World Health Organization (WHO) defines osteopenia as Bone Mineral Density (BMD)T-score of between -1 to-2.5. The WHO defines osteoporosis as BMD T-Score of lower than -2.5. (15]Because low bone mass can occur at a much younger age in thalassemia than in the general population, Z-score is used to assess bone mass in patients with thalassemia who are younger than 30 years old. Z score of lower than -2 considered as low BMD.

## Materials and Methods

In this cross sectional descriptive study, 10patients with transfusion dependent thalassemia younger than 18 years old attending to Motahari and Emam Khomeini hospitals in Urmia city were enrolled. Bone Mineral Density (BMD) was performed on an annual basis starting at around 10 years old. BMD was measured by a dual energy x-ray absorptiometry test that is commonly called a DEXA scan. 

**Statistical analysis**

The results of BMD measurement enable us to determine T-score or Z-score and to determine if they have osteopenia or osteoporosis.

## Results

10patients (6 male and 4 female) with transfusion dependent thalassemia (β-thalassemia major and intermedia) aged 13 to 17 years were enrolled. Mean age of patientswas15.1±.37year’s old and mean value of the height was 141±17.56 cm. Hemoglobin and ferritin level of patients were9.56±.47 g/dl and 1421.1±371.81 ng/ml. Among 10 patients, 8 patients (80 %) had low BMD and2 patients (20%) had normal BMD according to their Z-scoresin lumbar spine. Only 30% of patients had low BMD in the neck of femur (Table I).

**Table I:** Demographic features of patients

**Patient**	**Age**	**Gender**	**Height** **(cm)**	**Disease**	**Hemoglobin** **(gr/dl)**	**Ferritin** **(ng/ml)**	**Z score of lumbar** **spine**	**Z score of femoral neck**
**1**	15	male	137	Major thalassemia	9.2	1400	-2.2	-1.3
**2**	15	male	134	Major thalassemia	9.4	1020	-1.3	-1.2
**3**	16	male	138	Major thalassemia	10.2	1930	-3.0	-2.4
**4**	17	male	149	Major thalassemia	10.2	1700	-4.2	-2.2
** 5**	14	female	133	Thalassemia intermedia	9.4	1110	-2.0	-1.5
** 6**	15	male	138	Major thalassemia	9.3	930	-3.3	-3.0
**7**	13	female	135	Major thalassemia	10.2	1900	-0.8	-0.7
**8**	16	male	173	Major thalassemia	9.3	1200	-3	-1.3
**9**	16	female	139	Major thalassemia	10	1800	-2.5	-1.3
**10**	14	female	135	Major thalassemia	8.9	1256	-2.3	-1.8

## Discussion

Predisposing factors of osteoporosis in patients with transfusion related thalassemia are: endocrine insufficiency due to iron overload (delay in sexual maturation, hypoparathyroidism, hypothyroidism, diabetes mellitus and growth hormone insufficiency), direct iron toxicity on osteoblasts, progressive marrow expansion due to accelerated hematopoiesis and side effect of deferoxamine which is used as an iron chelator (1-5)

Wonke et al, studied thepolymorphism at the Sp1 location of the collagen type Ia1(COLIA 1) gene which is the major bone matrix protein and found that approximately 30% of the thalassemia major patients were heterozygotes and 4% were homozygotes for the Sp1 polymorphism. They concluded that men with thalassemia major carrying the Sp1 mutation may develop severe osteoporosis of the spine and the hip more frequently than patients who do not carry this mutation (16).

Detection of COLIA 1 polymorphism may have a role in identifying thalassemia patients that are susceptible to develop osteoporosis and pathologic fractures (17).

Voskaridou E, et al studied frequency of osteopenia or osteoporosis in well treated Thalassemia patients and found that approximately 40to 50% of them involved with this morbidity (1).

HaticeHamarat, et al studied frequency of osteoporosis in thalassemia major patients in Turkey and showed that among25 patients with thalassemia major (14 men, 11 women) 16 patients had osteoporosis, where as 9 patients had osteopenia (18).

Salim M AL Jadir, et al conducted a study on thalassemia patients and found that the prevalence of osteoporosis in thalassemia Iraqi patients was 67.5%, while osteopenia was found in 9.4% and normal BMD in 22.9% (19).

Karimi M, et al studied for Bone Mineral Density in Beta-Thalassemia Major and Intermedia and showed that Patients with thalassemia major and intermedia, younger than 20 yr., had lower BMD and BMC in the lumbar region (20).

In our study, 80%of transfusion dependent thalassemia patients in Urmia city had low BMD thus; we should aware and inform thalassemia patients about complications of osteoporosis such as bony fractures. Changing in life style is recommended to all transfusion dependent thalassemia patients with low BMD. 

## Conclusion

Among 10 patientsbelow than 18 y/o in Urmia city, 8 patients (80 %) had low BMD and 2 patients (20%) had normal BMD in lumbar spine according to their Z-scores. We should check transfusion dependent thalassemia patients with age more than 8 years old for bone loss by annual BMD and treat osteoporosis and osteopenia with administration of bisphosphonate, calcium and vitamin D supplements. Medical consultation with a rheumatologist and /or an endocrinologist should be performed in transfusion dependent thalassemia patients with bone loss. Changing of lifestyle with mild daily exercise and adequate Calcium rich foods (such as milk, yogurt and cheese, Dark green leafy vegetables such as broccoli, nuts, peas and baked beans) can prevent bone loss and fractures. Patients with osteoporosis should avoid heavy activities and stop smoking. Iron chelation therapy in adequate dosage can prevent iron toxicity on osteoblasts. Early diagnosis and treatment of endocrine insufficiencies can prevent bone loss. Regular blood transfusions help for prevention of progressive bone marrow expansion.
